# The IBD-FITT study — moderate-intensity exercise for patients with inflammatory bowel disease with moderate disease activity: an open-label randomized controlled trial

**DOI:** 10.1186/s13063-023-07781-4

**Published:** 2023-11-20

**Authors:** Ken Lund, Torben Knudsen, Jens Kjeldsen, Rasmus Gaardskær Nielsen, Carsten Bogh Juhl, Bente Mertz Nørgård

**Affiliations:** 1 Center for Clinical Epidemiology, Odense University Hospital, Odense, Denmark; 2 Research Unit of Clinical Epidemiology, Department of Clinical Research, University of Southern Denmark, Odense, Denmark; 3 Department of Medicine, Hospital of Southwest Jutland, Esbjerg, Denmark; 4 Department of Regional Health Science, Center Southwest Jutland, University of Southern Denmark, Esbjerg, Denmark; 5 Department of Medical Gastroenterology S, Odense University Hospital, Odense, Denmark; 6 Research Unit of Medical Gastroenterology, Department of Clinical Research, University of Southern Denmark, Odense, Denmark; 7 Hans Christian Andersen Children’s Hospital, Odense University Hospital, Odense, Denmark; 8 Research Unit of Pediatrics, Department of Clinical Research, University of Southern Denmark, Odense, Denmark; 9 Department of Sports Science and Clinical Biomechanics, University of Southern Denmark, Odense, Denmark; 10 Department of Physiotherapy and Occupational Therapy, University of Copenhagen, Herlev and Gentofte Hospital, Copenhagen, Denmark

**Keywords:** Inflammatory bowel disease, Crohn’s disease, Ulcerative colitis, Exercise

## Abstract

**Background:**

Inflammatory bowel disease (IBD), Crohn’s disease, and ulcerative colitis are chronic autoimmune lifelong diseases with fluctuating activity over time. The treatment includes medical therapy and surgery, however, there is no definite cure. Therefore, the quest for new and supplementary treatment options is imperative to improve patients’ general health and quality of life.

Physical activity and exercise have been suggested to be elements in both the prevention and supplementary treatment of IBD; however, this is based on limited underpowered trials. Thus, the role of exercise as a treatment option still has to be settled.

We aim to investigate the effect of a 12-week exercise intervention in adult patients with moderately active IBD on three categories of outcomes (1) disease-specific health-related quality of life (IBDQ); (2) general health status of the patients, i.e., waist circumference, disease activity by clinical scorings systems (Harvey Bradshaw Index, Simple Clinical Colitis Activity Index), blood pressure, blood lipids, and non-disease specific quality of life (EQ5D) scores; and (3) explorative outcomes on biomarkers (C-reactive protein and fecal calprotectin) plus different biomarkers of immunology (cytokine panel).

**Methods:**

We will apply a superiority design in this open-label randomized clinical trial including 150 patients equally allocated to intervention and usual care. The intervention will be based on a 12-week aerobic exercise program and will include two supervised exercise sessions of 60 min per week, combined with one weekly home training session. We have defined a moderate exercise level as 60–80% of patients’ maximum heart rate. The patients in the intervention group will also be offered an online video lesson of 15–25 min on lifestyle guidance, and the same online video lesson will be offered in the comparator group. Questionnaires on quality of life will be forwarded electronically both at inclusion and at the end of the study, and the patients will have blood samples, and fecal samples for calprotectin at baseline, weeks 4 and 8, as well as after 12 weeks (study end).

**Discussion:**

This will be a clinical trial investigating the effect of exercise on patients with Crohn’s disease and ulcerative colitis. This trial will add to the evidence on the possible effect of exercise and might clarify whether exercise can benefit as a supplementary treatment addendum. Thus, the trial may provide a new patient-active disease management approach.

**Trial registration:**

ClinicalTrials.gov NCT04816812. Date of first registration: March 23, 2021.

## Administrative information

Note: the numbers in curly brackets in this protocol refer to SPIRIT checklist item numbers. The order of the items has been modified to group similar items (see http://www.equator-network.org/reporting-guidelines/spirit-2013-statement-defining-standard-protocol-items-for-clinical-trials/).

**Table:** 

Title {1}	The IBD-FITT study — moderate-intensity exercise for patients with inflammatory bowel disease with moderate disease activity: an open-label randomized controlled trial
Trial registration {2a and 2b}.	Clinicaltrials.gov identifier: NCT04816812
Protocol version {3}	3 Feburary 2022, Version 2
Funding {4}	Financially supported by Pfizer, The Region of Southern Denmark, The Louis-Hansen Foundation, The Danish Colitis and Crohn's Foundation, The Research Council of Odense University Hospital, and The Tryg Foundation.
Author details {5a}	^1^Center for Clinical Epidemiology, Odense University Hospital, Odense, Denmark. ^2^ Research Unit of Clinical Epidemiology, Department of Clinical Research, University of Southern Denmark, Odense, Denmark. ^3^ Department of Medicine, Hospital of Southwest Jutland, Esbjerg, Denmark. ^4^ Department of Regional Health Science, Center Southwest Jutland, University of Southern Denmark, Esbjerg, Denmark. ^5^ Department of Medical Gastroenterology S, Odense University Hospital, Odense, Denmark. ^6^ Research Unit of Medical Gastroenterology, Department of Clinical Research, University of Southern Denmark, Odense, Denmark. ^7^ Hans Christian Andersen Children’s Hospital, Odense University Hospital, Odense, Denmark. ^8^ Research Unit of Pediatrics, Department of Clinical Research, University of Southern Denmark, Odense, Denmark. ^9^ Department of Sports Science and Clinical Biomechanics, University of Southern Denmark, Odense, Denmark ^10^ Department of Physiotherapy and Occupational Therapy, University of Copenhagen, Herlev and Gentofte Hospital, Copenhagen, Denmark
Name and contact information for the trial sponsor {5b}	Pfizer Inc. a Delaware corporation with an office of business at 235 East 42nd Street, New York, NY 10017 (“Pfizer”) The Region of Southern Denmark, Damhaven 12, 7100 Vejle, Denmark The Aage and Johanne Louis-Hansen Foundation, Gammel strandvej 22A, 2900 Nivae, Denmark The Danish Colitis and Crohn's Foundation, Nørregade 71-75, 1.th., 5000 Odense, Denmark The Tryg Foundation, Hummeltoftevej 49, 2830 Virum, Denmark
Role of sponsor {5c}	Pfizer has awarded a fellowship grant (financial) to the IBD-FITT study based upon the study protocol. Pfizer is not involved in any part of the study and has no ultimate authority over any of the scientific activities. The Region of Southern Denmark, The Louis-Hansen Foundation, The Danish Colitis and Crohn’s Foundation, The Research Council of Odense University Hospital, and The Tryg Foundation have awarded funding (financial) to the IBD-FITT study based upon the study protocol. The funding parties are not involved in any part of the study and have no ultimate authority over any of the scientific activities.

## Introduction

### Background and rationale {6a}

Inflammatory bowel disease (IBD) includes Crohn’s disease and ulcerative colitis. The diseases are chronic autoimmune lifelong diseases with fluctuating disease activity over time [1, 2]. The incidences of Crohn’s disease and ulcerative colitis are increasing with approximately 60,000 patients suffering from the diseases in 2021 [1, 2]. The etiology and development of the diseases are considered to be a mixture of environmental, genetic, and behavioral factors [3, 4]. The chronic inflammation can be located in the entire gastrointestinal tract for Crohn’s disease and located in the large intestines for ulcerative colitis with common symptoms such as pain, fever, and diarrhea [3, 4].

The treatment is complex and includes medical therapy with corticosteroids, immunomodulators, biologics, and surgery, but there is no definite cure [3, 4]. Therefore, the quest for new and supplementary add-on treatment options is imperative to improve the patient’s general health and quality of life, reduce inflammation, and counteract the adverse effects of the treatment.

Lower levels of physical activity have been observed in patients with IBD compared to healthy persons [5, 6], but the role of exercise as a treatment option still has to be settled. Physical activity and exercise have been suggested to be important and safe elements in the prevention and as a supplementary option in patients with IBD, but these suggestions are only based upon limited underpowered trials [7–14]. These studies are hampered by different study populations with the inclusion of patients with different levels of disease activity at baseline, different types and intensities of physical activity plus a variety of different outcomes. However, several reviews have suggested different theoretical beneficial effects of exercise in patients with IBD [6, 15–21]. Exercise is a well-known supplementary treatment modality in other chronic diseases such as cardiovascular disease, diabetes, and rheumatoid arthritis where exercise improves general health and positively influences the quality of life. The mechanisms by which exercise could have a positive impact on patients with IBD may be linked to the direct anti-inflammatory response after moderate exercise intensities [6, 15, 16, 19, 20]. Moreover, exercise could also reduce fatty tissue in the abdomen and intestine in relation to overweight, [22] restore or increase muscle mass, positively influence the repair process of mucosal damage, increase bone health, and prevent osteoporosis [17]. Reduced muscle mass and osteopenia are well-known conditions in patients with IBD and may be related to the chronic inflammatory state/persistent inflammatory activity or malabsorption of micro and macro nutrients [17, 20, 23]. Furthermore, inactivity is especially known to be linked to heart disease and increased mortality in the general population, and to some extent, obesity and increased waist circumference are known risk factors as well [24–28].

This study will be a clinical interventional trial of the effect of exercise in patients with Crohn’s disease and ulcerative colitis. We aim to investigate the effect of exercise in adult patients with moderately active disease on three categories of outcomes (1) health-related quality of life (Inflammatory Bowel Disease Questionnaire (IBDQ)); (2) general health status of the patients such as waist circumference, disease activity by clinical scoring systems used in IBD (Harvey Bradshaw Index (HBI), Simple Clinical Colitis Activity Index (SSCAI), blood pressure, blood lipids, and non-disease specific quality of life scores (EQ5D); and (3) explorative outcomes including biomarkers of C-reactive protein and fecal calprotectin plus different biomarkers of immunology (cytokine panel). These outcomes will be examined after 12 weeks of the exercise intervention.

This study will contribute to clarify the possible effects of exercise with a properly sized clinical trial and might clarify whether exercise can benefit as a treatment addendum. The study might thus provide a new active patient disease management approach for patients with IBD.

### Objectives {7}

In adult patients with moderately active ulcerative colitis and Crohn’s disease, we want to investigate whether exercise therapy during 12 weeks including a lesson on generally healthy lifestyle is more effective compared to control patients only receiving a lesson on generally healthy lifestyle recommendations on the outcomes of interest.

### Trial design {8}

**Fig. 1:**
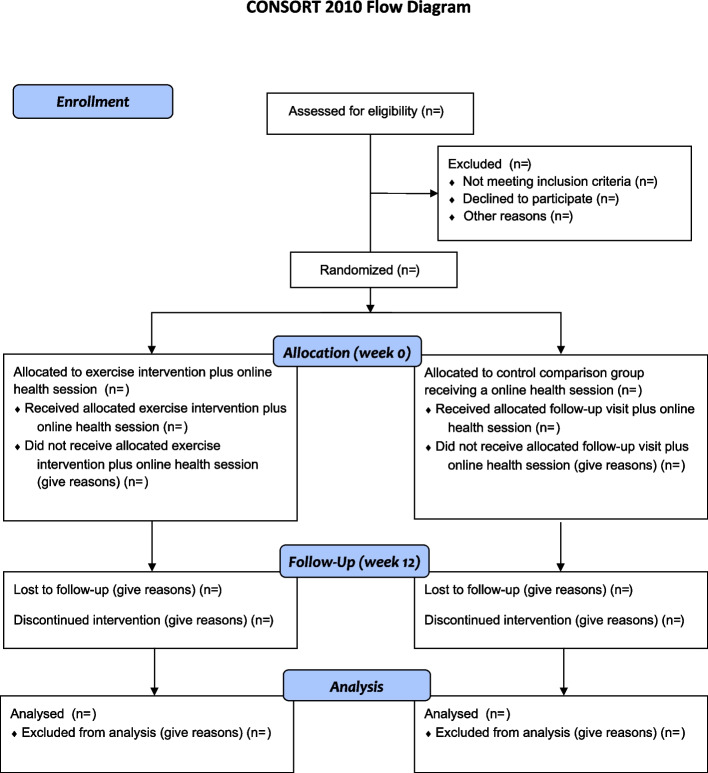
Study design

We will apply a superior trial design in this open-label randomized clinical trial with two arms, intervention, and comparison (see Fig. 1). We thus want to assess whether the intervention is better than (superiority trial) a comparator [29, 30].

## Methods: participants, interventions, and outcomes

### Study setting {9}

We will recruit patients with Crohn’s disease or ulcerative colitis from specialized gastroenterology out-patient clinics located at two separate hospitals:

1. Department of Medical Gastroenterology, Odense University Hospital, Odense, Denmark
2. Department of Medicine, Hospital of Southwest Jutland, Esbjerg, Denmark

### Eligibility criteria {10}

#### Inclusion criteria

- Male or female aged between 18 and 65 years (included)
- Well-characterized Crohn’s disease or ulcerative colitis (physician assessment):
  - ◦ Fulfilling standard clinical, endoscopic, radiologic, and histological criteria according to clinical guidelines
- A minimum of 1 year of disease duration from the time of diagnosis to the inclusion
- One or more of the following pharmacological treatments within 3 months before inclusion:
  - ◦ 5-Aminosalicylic acid
  - ◦ Corticosteroids
  - ◦ Azathioprine, 6-mercaptopurine, methotrexate
  - ◦ Anti-TNFα (infliximab, adalimumab, golimumab), ustekinumab, or anti-integrin (vedolizumab)
- Disease activity defined by clinical disease score:
  - ◦ HBI [31] > 5 [32]
  - ◦ SCCAI [33] > 5 [32, 34]
- Marker for gastrointestinal inflammation:
  - ◦ Fecal calprotectin > 200 mg/g [32] or C-reactive protein ≥ 6
- The patient must be medically evaluated for safe participation in the intervention by the physician in charge of the patient

#### Exclusion criteria

- A language barrier that prohibits the exercise instructors/supervisors to provide sufficient or safe instructions during the intervention or at the general health lesson
- A known heart condition that prevents patients from actively participating safely in the exercise intervention
- Known pregnancy, planned pregnancy, or pregnancy during the intervention period because pregnancy needs extra precautions in the pharmacological treatment, and exercise with high intensity is not recommended limiting these patients to participate in the exercise intervention
- Disabling musculoskeletal injuries that limit patients from actively participating in the exercise intervention
- Severe disease activity manifested clinically and with the need for urgent change in medical or surgical treatment strategy evaluated by the responsible physician

The responsible physician at the respective department of gastroenterology screens through the medical records for eligible patients. The screening information is not passed on to the research project, except for the personal identifier of patients and telephone number which are passed on to the research nurse. The eligible patients are approached at their upcoming appointment with information and the possibility to participate in the trial by the physician or research nurse. This is coordinated with the research nurse to ensure only eligible patients are approached at their forthcoming appointment. At each outpatient clinic, written information and posters will be used to inform patients of the trial and create awareness for the patients on the possibility to enter the trial.

### Who will take informed consent? {26a}

We will use a consecutive inclusion of patients who fulfill the defined inclusion criteria. We plan with a recruitment period of 1–2 years. Patients will receive oral and written information on the current research project from the specialized health personnel, either physician or research nurse, in a quiet setting. After information, the patients are given at least 1 day of the consideration time, to determine whether or not they would be interested in participating and then give informed consent. If the patient wishes to have a relative present when receiving the information, this is encouraged and will be arranged at the study site. A research coordinator (nurse) will contact the patient who has accepted to be contacted after 1–3 days by telephone to confirm the acceptance of participation in the project. The patient will hereafter be asked to return a signed written pre-filled consent form.

### Additional consent provisions for collection and use of participant data and biological specimens {26b}

After obtaining written consent from the patients, the different eligibility criteria are recorded in the study’s research database (Redcap database hosted by Odense Patient Exploring Network (OPEN) situated in Denmark). This includes information on diagnosis, the onset of disease, duration of disease, disease activity by clinical scores and fecal plus blood biomarkers, and type of pharmacological treatment 3 months before inclusion. The written consent includes a permission to store blood samples and fecal samples in a research biobank also hosted by OPEN. The written consent includes a permission for the research group and regulatory authority to obtain health status from the medical record to monitor the quality and safety of the intervention.

## Interventions

### Explanation for the choice of comparators {6b}

We include patients with Crohn’s disease and ulcerative colitis with moderate disease activity as comparators and offer them one online lesson of 15–25 min on lifestyle guidance including general advice on healthy living. Specialized health personnel will present an educational video lesson. The patients in the comparison arm will receive the usual medical treatment with regular follow-up in the outpatient clinic. In this group, we will also monitor 7 days of physical activity level by accelerometry using the AX3 accelerometer [Axivity Ltd., Newcastle upon Tyne, UK] in weeks 0 and 12. The comparators have been chosen to represent the usual treatment and to help determine the efficacy of the intervention. Furthermore, all patients in the comparison arm will receive a wrist-worn heart rate monitor/fitness tracker (commercial) after completing a follow-up program identical to the patients in the intervention arm.

### Intervention description {11a}

The intervention is based on a 12-week aerobic exercise program tailored to the individual patients by physiotherapists using the principles of frequency, intensity, time, and type (FITT) aiming to increase or maintain the physical activity level to a weekly moderate level (metabolic equivalent of a task (MET) ≥ 3) [26]. This includes two supervised exercise sessions per week, each session with a length of 60 min combined with one weekly home training session. We have defined a moderate exercise level as 60–80% of their maximum heart rate, and the average intensity is calculated over the active part of the supervised session. The two weekly 60-min sessions will be supervised and the type of exercise is individually tailored by a physiotherapist to fit each patient.

The patients in the intervention arm will also be offered an online video lesson of 15–25 min on lifestyle guidance including general advice on healthy living by behavioral issues similar to the comparators. The patient will receive the usual medical treatment throughout the intervention.

The exercise intervention and home training are monitored by training diaries including frequency, intensity, time, and type of exercise. We will use the BORG 15-point scale (rate of perceived exertion) and heart rate monitors (commercial) to monitor intensity in-session [35]. We will use a heart rate monitor to quantify the cardiovascular intensity by heartbeat measure and to guide the patients during aerobic exercise to reach a moderate activity level of exercise. We use the maximum heart rate to calculate the target moderate exercise level of 60-80% and we will establish the maximum heart rate at the first supervised exercise session using a maximum cycling exercise protocol (Watt-max cycling test) or use an estimated heart rate (211–0.64*age) if the cycling can not be carried out by the participant [36, 37]. The training diary is used throughout the 12 weeks of intervention also to increase compliance/adherence and to allow proper feedback to the patients on their progress. We will also monitor the overall 7 days’ physical activity level by accelerometry using the AX3 device from the company Axivity in weeks 0 and 12.

There is solid evidence to support the appropriateness of a 12-week follow-up period when it comes to the quality of life endpoint [21, 38, 39], waist circumference [39], disease activity [21], blood pressure [21, 39], and blood lipids [21, 39]. Physical activity and exercise in patients with IBD and low to moderate disease activity have previously been found to be safe and no provoked disease activity has been observed [6, 9, 17]. The intervention will take place in-hospital at Southwest Jutland Hospital and at a private authorized physiotherapist clinic.

### Criteria for discontinuing or modifying allocated interventions {11b}

There are no specific cut-off criteria set up for discontinuing the intervention, however, if a patient experiences a significant increase in clinical disease activity or similar issues and the responsible physician estimates it as significant, the participation in the trial is discontinued.

### Strategies to improve adherence to interventions {11c}

We have applied several strategies to improve adherence to the intervention. We will have supervised exercise sessions, and use exercise diaries to track the patient progress. The patients can keep the wrist-worn heart rate monitor/fitness tracker at the end of the study as a gift for completing follow-up.

### Relevant concomitant care permitted or prohibited during the trial {11d}

The patients enrolled in the study will receive relevant concomitant care during the entire study period regardless of being randomized to the interventional arm or comparator arm.

### Provisions for post-trial care {30}

All participants included in the study will be informed of the patient's rights when included in a trial. In case of any unintended harm to the participants in the study during the intervention, they will have the right to compensation defined by Danish legislation (Patienterstatningsordningen). Patients may choose to withdraw at any time.

### Outcomes {12}

The primary outcome was chosen in collaboration with our patient representatives connected to this study, and because the health-related quality of life is a multidimensional measure incorporating the patient life situation. The secondary outcomes were chosen to reflect the overall general health status of the patient as well as the non-disease specific quality of life, where the explorative outcomes focus on changes in the immune system.

### Primary outcome

We will use the Danish version of the IBDQ [40].

1. IBDQ

### Secondary outcomes

1. Waist circumferences
2. Clinical disease activity:
  i. HBI
  ii. SSCAI
3. Blood pressure
4. Lipid status:
  i. Low-density lipoprotein (*LDL)*
  ii. High-density lipoprotein (*HDL)*
  iii. Triglycerides
  iv. Total cholesterol
    1. Total cholesterol = LDL+ HDL + (20% of the total triglycerides)
  v. Hemoglobin A1c
5. EQ5D-5L (not disease-specific) [41]

#### Explorative outcomes

1. Biomarkers:
  i. Fecal calprotectin
  ii. C-reactive protein
2. Immunology, biomarkers (Panel - cytokines):
  i. Interleukin 6
  ii. Interleukin 8
  iii. Interleukin 10
  iv. Tumor necrosis factor alpha

The outcomes regarding the quality of life are measured at baseline and week 12, whereas the disease scores and biomarkers are measured in weeks 0, 4, 8, and 12. We will analyze blood and stool samples at baseline, 4, 8, and 12 weeks. The analyses are carried out within 5–7 days after the samples are collected, except for the analysis of cytokines which are analyzed at the end of the study.

### Participant timeline {13}

**Table:** 

**Timepoints**	**Study period**				
	**− t1**	**t0**	**t1**	**t2**	**t3**
	**Pre-study**	**0**	**4 weeks**	**8 weeks**	**12 weeks**
**Eligibility** Pre-screening	X				
Inclusion/exclusion	X				
Informed consent	X				
Allocation (randomization)	X				
**Outcome** *General health* Waist circumference Blood pressure Disease activity (HBI + SSCAI)		X X X			X X X
*Blood and stool samples* C-reactive protein Fecal calprotectin Lipid status Cytokines		X X X X	X X X X	X X X X	X X X X
*Health-related quality of life* IBDQ EQ5D		X X			X X
*Activity monitoring* Accelerometer (AX3)	X				X
**Interventions** Exercise intervention		X	X	X	X
Online lesson		X			

#### Sample size {14}

To calculate the number of patients needed in this trial, we have used a power of 80% and an alpha level of 5% (0.05) and used outcome differences observed in previous studies. We need to include at least 106 patients, with 53 patients in the intervention arm and 53 in the comparison arm to be able to detect a clinically relevant 13.8 (SD ± 25) mean difference in change between the groups on the IBDQ scale based on the similar changes observed in studies by Ng, Millard [12] and Klare, Nigg [9]. A minimum clinical important difference (MCID) for the IBDQ has previously been reported as an absolute change of 16 points in patients with Crohn’s disease; however, MCID for the IBDQ is not clearly defined [42]. We have performed a power analysis on the secondary and explorative outcomes, not reported, and overall we need to include a total of 100 patients in these analyses. Based on this, and to cover all outcomes including 33% drop-out, we have estimated a total study size of 150 patients, with 75 in both the intervention arm and the comparator arm.

### Recruitment {15}

We will use consecutive enrollment of patients at two large study sites to help achieving adequate participant enrolment over an estimated period of 1 year. However, we can prolong the inclusion period if necessary. We will use roll-ups and flyers for awareness of the study towards the patients at each site.

## Assignment of interventions: allocation

### Sequence generation {16a}

We will use a 1:1 computer-based randomization module embedded in RedCap provided by OPEN using computer-generated random numbers and in blocks of four.

### Concealment mechanism {16b}

The research nurses do not have any access to the random list for the allocation of patients, because the list is incorporated in the randomization module and only a randomization button will appear in the system.

### Implementation {16c}

The research nurse will generate the randomization at inclusion and according to the randomization, patients will be assigned to either the intervention arm or the comparison arm.

## Assignment of interventions: blinding

### Who will be blinded {17a}

This study includes an exercise intervention that does not allow for the blinding of the participants or the healthcare personnel involved in the study.

### Procedure for unblinding if needed {17b}

No procedure for unblinding.

## Data collection and management

### Plans for assessment and collection of outcomes {18a}

We will create a research database hosted by OPEN in Odense, Denmark to enter and store the data, as this solution applies to the General Data Protection Regulation and Danish legislation to securely handle patient data. The research database is based on the system by Redcap and the database is available by any internet browser, allowing the sites to enter data directly into the database.

We will store the data for up to 5 years after the end of the project, and hereafter the data will be either transferred to the Danish National Archives and made anonymous, or destroyed.

Patients that are included in the study will have to have a blood sample (approximately 11.3 ml per patient) and a small fecal sample (approximately 2 × 3 ml per patient). The sample material and any excess material will be deposited in a research biobank after analyses during the project. The material in the research biobank will after the end of the project be stored for up to 5 years in a future biobank enabling any future research in relation to this project, and both biobanks follow the valid general data protection regulation. Patients also need to have external measures, such as waist circumference, blood pressure, and answer questionnaires regarding quality of life.

### Plans to promote participant retention and complete follow-up {18b}

The participating patients will be able to receive economical compensation for transportation expenses for the ambulatory visits. In case of withdrawal during follow-up in the intervention group, we will ask the patients whether they still could be interested in answering the electronic questionnaires or completing the ambulatory visits to ensure follow-up and likewise in the comparative group. However, the patients may at any time withdraw their consent to participate without any influence on their medical treatment.

### Data management {19}

The data entry is completed at the two sites by the research nurses and research physiotherapists using the electronic research database (RedCap). The database has incorporated range checks for numeric data, and the data will be checked by the principal investigator during the entire study period. The electronic surveys are directly sent to each patient’s e-box. All Danish citizens have an electronic e-box for communication with the state, and the e-box is linked to their civil registration number. The principal investigator manages access to the database. The accelerometers are sent in pre-paid envelopes from the participants to the principal investigator, where the data is loaded into a secured storage space hosted by OPEN. The database and storage use logging and follow the EU GDPR rules.

### Confidentiality {27}

All the personal information is stored in the research database during the study and up to 5 years after. Only the principal investigator and the research group have access to the data, and the data may not be used for any other purposes than described and approved by the regional ethics committee. The data must not be shared with others to protect the confidentiality of the participants, and data sharing will require new consent from the patients.

### Plans for collection, laboratory evaluation, and storage of biological specimens for genetic or molecular analysis in this trial/future use {33}

The blood samples and fecal samples are stored in a research biobank hosted by OPEN. The specimens are saved for 5 years in the biobank after the end of the study. All new studies that will use data must be approved by the local ethics committee, as well as the research group including the principal investigator.

## Statistical methods

### Statistical methods for primary and secondary outcomes {20a}

We will present descriptive statistics using absolute numbers and percentages. We will use the intention-to-treat (ITT) concept in our primary analysis of the specified outcomes on between-group differences from baseline to week 12. We will use parametric or non-parametric statistics for comparing the primary outcome in the interventional arm to the comparison arm. We use a *t*-test if the data is parametric and a Wilcoxon rank-sum test if the data is non-parametric to estimate the average causal effect of the intervention. We will use mixed model analyses with random effects to evaluate repeated outcome measurements at weeks 0, 4, 8, and 12, and to account for within-patient effects on our outcome measures opting for more precise estimates of treatment effects [43]. In a supportive analysis of the data, we will use a per-protocol (PP) strategy for handling protocol non-adherence. We will report the 95% confidence intervals for the average causal effects estimates. We will report our results according to the CONSORT statement for randomized clinical trials [44]. We will use STATA to process data and calculate the estimates.

### Interim analyses {21b}

There is no planned interim analysis, due to the short study period of 12 weeks. However, we will monitor any adverse events in relation to the intervention.

### Methods for additional analyses (e.g., subgroup analyses) {20b}

We will perform adjusted and stratified analyses including mixed-effect models to examine other plausible differences, for example, differences in average causal treatment effect between men and women, and adjusting for site. We plan to explore differences in stratified analyses based on the patient type of disease, disease severity at baseline, type of pharmacological treatment at baseline, and physical activity level measured by accelerometry at baseline.

### Methods in analysis to handle protocol non-adherence and any statistical methods to handle missing data {20c}

We will use a PP approach for handling non-adherence and examine the robustness of the ITT analysis results. In the PP analysis, we will include patients based on their adherence to the protocol considered as complete cases (e.g., patients with more than 80% show up in the intervention arm and complete follow-up in the comparison arm) and to analyze the specified outcomes on between-group differences from baseline to week 12 for this population. As specified earlier, we apply similar mixed model analyses in the PP approach as in the ITT. In supplement to the PP analyses and the possible non-adherence, we plan to use causal inference methods that include emulating data, for example, effects of different types of non-adherence or effects of concomitant treatment utilizing marginal structured models and inverted probability weighting to estimate the average treatment effect under these explorative additional questions [45, 46].

In the case of missing data in the ITT analysis, we will use multiple imputations with 10 imputations per missing value if the data is assumed to be missing at random or not missing at random. The multiple imputations will be based on regression models including predictive covariates and/or outcomes depending on which data are missing. We plan to use a linear model for continuous outcomes including covariates such as age, sex, type of disease, and disease activity. If data is assumed not to be missing at random, we will attempt to understand why and report the differences in our results by the complete-case approach and when imputation analyses are applied. To deal with any covariates or outcomes that are not normally distributed we will use log or other appropriate transformations [47]. We will perform sensitivity analysis with a best/worst approach to missing data as well as helping evaluate the robustness of the analysis.

### Plans to give access to the full protocol, participant-level data, and statistical code {31c}

There is no current plan to give access to the full protocol, participant-level data, or the statistical code, however, the results will be published in peer-reviewed journals that may allow for statistical codes as supplementary.

## Oversight and monitoring

### Composition of the coordinating center and trial steering committee {5d}

The data will be monitored by the principal investigators, and access to the database is also handled by the principal investigator in collaboration with a data manager located in the Research Unit for Clinical Epidemiology, University of Southern Denmark, Odense, Denmark. The entire research group has access to the raw data. The research group will meet at least quarterly during the 1–2-year inclusion period and meetings will also be arranged on an ad hoc basis during the study period. The trial protocol has been registered at Clinical Trials (ClinicalTrials.gov — identifier: NCT04816812) after ethics approval was obtained.

### Composition of the data monitoring committee, its role and reporting structure {21a}

No data monitoring committee is set up for this trial. The data monitoring is done by the primary investigator and is independent of the sponsors. In Denmark, only trials approved by the Danish Medicines Agency, to investigate new drugs or changes in drugs, are mandatory to be monitored by The Regional GCP units (Good-Clinical-Pratice Units) in accordance with Danish legislation. This current trial on exercise does not involve any change in the patient's pharmacological treatment.

### Adverse event reporting and harms {22}

Adverse events are defined as side effects that are harmful due to the exercise intervention, e.g., severe musculoskeletal injuries that prevent participation or major increases in clinical symptoms related to IBD needing medical attention [48]. The included patients with IBD are considered to be at equal risk of any musculoskeletal injury as healthy patients start to exercise, and the exercise sessions will be supervised by trained health personnel. If patients in the project experience any adverse effects or harms due to the intervention or the study, the patient will be referred to the emergency department or the treating specialist physician at the gastroenterology department for an assessment and further treatment. All adverse events or harms are reported and recorded either by the research nurse or the physiotherapist during each exercise session in the research database. In case of any severe adverse event (SAE), e.g., potential life-threatening fall, the health professional will contact the emergency department/service as described by local in-hospital guidelines or by regular emergency service outside the hospital.

### Frequency and plans for auditing trial conduct {23}

We plan to audit the trial data every month. The primary investigator and one member of the research group will oversee the data including any adverse events to be reported.

### Plans for communicating important protocol amendments to relevant parties (e.g., trial participants, ethical committees) {25}

All modifications of the protocol will be reported to the regional ethics committees, and all changes will be reported in the participation information. The principal investigator is responsible for updating the online registration on trials.gov.

### Dissemination plans {31a}

We will publish the data in peer-reviewed scientific journals, present at international research conferences, and reach out to local patient associations.

## Discussion

This trial is the first trial trying to include a large number (*n* = 150) of patients with IBD and moderate disease activity in order to explore the effect of an exercise intervention. We have chosen this number of patients to be able to evaluate meaningful effects in our specified outcomes. We expect that the results will be helpful in future clinical guidelines, and add highly relevant knowledge on the effect of exercise that may guide patients in clinical practice and may provide a new patient-active disease management approach. The current evidence on the effects of exercise on IBD is inconsistent with few clinical trials with a sufficient number of included patients.

The randomized controlled study design applied in our trial is a strength. Another strength related to this trial is that the protocol has been developed with guidelines from the SPIRIT and PREPARE guidelines, [30, 49] and that the protocol is being published to enhance clearance and transparency. Further strengths in the development of the protocol are that patient representatives were involved to help inform plus guide the selection of outcome of interest, design exercise regime using the FITT principles, [26] and online lifestyle presentation. Another strength is that the measurements in the trial are based on validated questionnaires in combination with objective repeated measurements, e.g., accelerometry, blood samples, and fecal samples that are stored in a biobank to evoke current and future relevant research.

Our trial has some limitations. Due to the nature of the exercise intervention blinding the research nurses, physiotherapists, and participants is not an option. Limitations according to the recruitment of participants do exist because we focus on patients with mild to moderate disease activity. These patients may be more cautious to participate due to symptoms or because of the possibility of not being randomized to the intervention or not being able to participate in the intervention because of their schedule. Another limitation of the trial is that we have not planned follow-up after week 12. However, we have chosen not to plan a long-term follow-up because we do not yet clearly know the short-term effect of exercise, which is the focus of this trial. Selection bias may be of concern when conducting a trial on exercise, and this may influence the generalizability of the study.

## Trial status

Protocol version 4 — 25 November 2021. Recruitment started on 15 November 2021 and approximately ends on 15 November 2022. Inclusion extended to 1 May 2023.

## Data Availability

The principal investigator and the research group will have access to the final trial dataset. Access to the data is limited by Danish legislation, and patient data may not be directly available to other parties. The research group does not have any plans to share data currently.

## Abbreviations

GCP: Good Clinical Practice
HBI: Harvey Bradshaw Index
IBD: Inflammatory bowel disease
IBDQ: Inflammatory Bowel Disease Questionnaire
ITT: Intention-To-Treat
MCID: Minimum Clinical Important Difference
PP: Per-Protocol
SCCAI: Simple Clinical Colitis Activity Index
